# Genomic diversity of vaccinia virus strain Cantagalo isolated in southeastern Brazil during the early years of the outbreak, 1999-2006

**DOI:** 10.1590/0074-02760200521

**Published:** 2021-02-03

**Authors:** Aline RV Souza, Matheus Nobrega Luques, Clarissa R Damaso

**Affiliations:** 1 Universidade Federal do Rio de Janeiro, Instituto de Biofísica Carlos Chagas Filho, Rio de Janeiro, RJ, Brasil

**Keywords:** virus diversity, poxvirus, zoonosis, Cantagalo virus

## Abstract

Outbreaks of a vesiculopustular disease in dairy cattle and milkers have been frequently reported in Brazil since 1999 when the vaccinia virus strain Cantagalo was first isolated in the State of Rio de Janeiro. However, the genomic diversity of the viral isolates associated with these outbreaks is not well known, particularly in the southeastern states that represent the focal point of virus spread to other regions. Here, we report the genomic sequences and an analysis of the polymorphic site profiles and genotypic diversity of four clinical isolates of vaccinia virus strain Cantagalo collected from 1999 to 2006 in southeastern Brazil.

Vaccinia virus (VACV) is a large and complex double-stranded DNA-containing virus that belongs to the family *Poxviridae*.^1^ Different strains have been used as the smallpox vaccine until the early 1980s after the declaration of smallpox eradication.^2^ Despite its worldwide use as a vaccine, VACV does not cause natural infections in humans and animals, except in India, Colombia, and Brazil.^3^ ^,^ ^4^ ^,^ ^5^

In Brazil, outbreaks of a vesiculopustular disease in dairy cattle and milkers have been frequently reported since 1999.^4^ The first case was reported in farms in the State of Rio de Janeiro (RJ) and led to the isolation of a novel strain of VACV that was named Cantagalo virus (CTGV).^6^ A similar virus was also isolated in the State of São Paulo in the same year and it was named Araçatuba virus.^7^ The disease causes localised vesiculopustular lesions on the teats and udder of dairy cows and on the hands and arms of milkers, accompanied by high fever, malaise, axillary lymphadenopathy, and occasional secondary bacterial infection on the lesion sites. Infected cows cannot be milked for a couple of weeks and may develop secondary mastitis, generating important economic losses, particularly in non-automated farms.^8^ Milkers are infected occupationally and the infection is usually cleared in approximately three weeks, but occasional complications have been reported.^9^ ^,^ ^10^

After the first outbreaks in 1999, the number of reported cases increased with the rapid spread of the infection to the other southeastern states, i.e., Minas Gerais (MG) and Espírito Santo (ES).^10^ ^,^ ^11^ ^,^ ^12^ ^,^ ^13^ ^,^ ^14^ ^,^ ^15^ ^,^ ^16^ From there, the virus disseminated to other regions of the country, mainly in association with the animal trade.^17^ ^,^ ^18^ ^,^ ^19^ ^,^ ^20^ ^,^ ^21^ Most studies have addressed the epidemiological and clinical aspects of the outbreaks with limited genetic analyses of the virus isolates. At present, only two genomes have been fully sequenced and properly assembled: the genome of the first CTGV isolate CM01^22^ and the genome of Serro2 virus, a VACV isolate similar to CTGV and Araçatuba, obtained from human lesions in MG in 2005.^10^ ^,^ ^15^ Thus, despite the numerous reports of VACV outbreaks in Brazil during the last twenty years, little is known about the genomic diversity of the clinical isolates. Of particular relevance, we highlight the poor knowledge of the VACV genomes associated with the early episodes of infection in the states of the southeastern region, which played a major role as a focal point of virus spread to other regions of Brazil. Therefore, in this work, we sequenced and analysed the full genome of four clinical isolates of CTGV collected from 2000 to 2006 in the southeastern states of RJ, MG, and ES.

Outbreaks of vesiculopustular disease affecting dairy cattle were previously reported by the state secretariats for animal health surveillance and their affiliated agencies in the municipalities of Miracema (RJ), Vieiras (MG), Campos de Goytacazes (RJ), and Alegre (ES) in 1999, 2001, 2003, and 2006, respectively (Fig. 1). Scab samples sent to our laboratory tested positive for CTGV by polymerase chain reaction (PCR) diagnostics based on the A56R gene, as previously reported.^11^ For genome sequencing, viral crude stocks were submitted to one round of plaque-purification, and representative plaques were isolated for virus propagation in BSC-40 cells, followed by ultracentrifugation in 25-45% potassium tartrate gradients. Genomic DNA from purified virus particles was isolated using the Wizard® Genomic DNA Purification Kit (Promega, Madison, WI), as previously described.^22^ Libraries were prepared using the Illumina genomic Nano kit, followed by 2×150+8 paired-ended sequencing runs using a NextSeq 500 platform (Illumina, San Diego, CA, United States). Quality-trimmed and adaptor-removed raw reads (30.971.724 to 35.756.458) were used for *de novo* assembly using Velvet vs 1.2.10 with a kmer of 149.^22^ ^,^ ^23^ The resulting contigs were used as input for SeqMan (DNAStar Lasergene 15) for proper assembly of the inverted terminal repeats (ITRs).^24^ Assemblies were inspected for quality and removal of ambiguities by mapping the raw reads to the final contig using BWA version 0.7.12 (http://bio-bwa.sourceforge.net/), followed by visualisation in Tablet 1.17.08.17.^22^ Genomes were initially annotated based on a reference genome (the CTGV-CM01 Genbank accession number KT013210) using GATU software (https://4virology.net/), followed by manual editing using CLC Main Workbench vs 7.9.1 to call new open reading frames (ORFs) that were Blastp-confirmed.^22^ The program Mafft 7.0 (https://mafft.cbrc.jp/alignment/server/) was used for all multialignment analyses using default parameters.

**Fig. 1:**
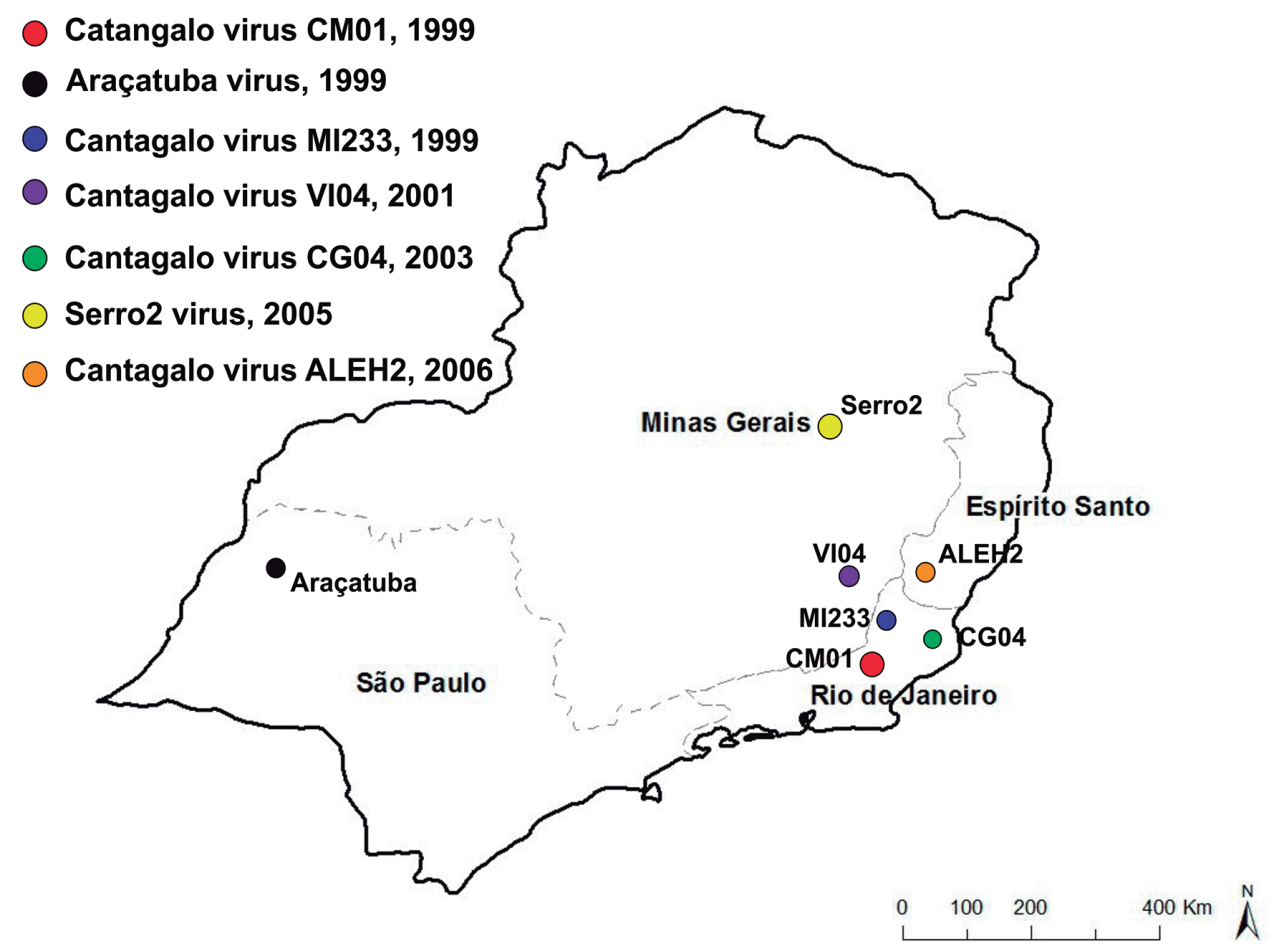
schematic map of the sites and dates of sample collection in the southeast region. In 1999, two outbreaks of vaccinia virus (VACV) infection in dairy cattle were reported in the State of Rio de Janeiro (Cantagalo virus isolate CM01) and in the State of São Paulo (Araçatuba virus). Other outbreaks were reported in subsequent years, but only the location of outbreaks caused by Cantagalo virus (CTGV) isolates MI233, VI-04, CG04, and ALEH2 are shown. The locations where Serro2 virus and CTGV-CM01 were collected, as well as Araçatuba virus, are also indicated together with the date of collection.

The Table summarises the sequencing data and statistics obtained for the four genomes. Genome sizes were very similar among all isolates, and the same was observed for the ITR, which are terminal regions repeated in both ends but in opposite directions. However, the ITR sizes were much smaller than the average size of ITRs found in other VACV strains, usually longer than 10 kb.^22^ ^,^ ^25^ These are important diploid regions of the genome bearing two copies of several virulence genes.^1^ Genome identity scores (Table) and phylogenetic inference (Fig. 2) confirmed the classification of the four samples as CTGV isolates, previously determined by PCR diagnostics.^11^ Based on the analysis of sequence identity (Table), CTGV-CG04 should be considered the closest isolate to CTGV-CM01. However, this suggestion is not supported by the analyses of the number of single-nucleotide polymorphisms (SNPs) (Table), the phylogenetic tree (Fig. 1), and, in more detail, by the median-joining network (Fig. 3). Taken together, they point to CTGV-MI233 as the closest isolate to CTGV-CM01, followed by isolates VI04 and CG04. Because such methods do not take into account insertions and deletions (INDELs), a 89-bp insertion that CTGV-MI233 has within the hypervariable region of the A51R gene relative to CTGV-CM01 was not considered in the analyses. Within the same genomic region, VI04 and CG04 isolates have 61-bp and 8-bp insertions, respectively, when compared with CTGV-CM01 (Fig. 4, asterisk). Still, regardless of the method of analysis, all three isolates were > 99.9% similar to CTGV-CM01 (Table), which is somewhat expected because of the close collection sites and dates (Fig. 1).

**TABLE:** Sequencing statistics and genomic features of the Cantagalo virus (CTGV) isolates MI233, VI04, CG04, and ALEH2

Sample	City/State/year	Genome size (bp)	ITR size	No. ORFs	Genome coverage^*a*^	No. SNPs^*b*^	% identity^*c*^	Genbank accession
CTGV-MI233	Miracema (RJ), 1999	181,064	7,025	218	26,098 x	2 (41)	99.94 (97.10)	MW018153
CTGV-VI04	Vieiras (MG), 2001	181,894	7,393	219	27,637 x	13 (52)	99.95 (97.10)	MW018154
CTGV-CG04	Campos dos Goytacazes (RJ), 2003	180,989	7,024	223	26,600 x	14 (53)	99.98 (97.13)	MW018155
CTGV-ALEH2	Alegre (ES), 2006	184,841	7,023	225	23,493 x	62 (81)	97.86 (99.09)	MW018156
CTGV-CM01	Cantagalo (RJ), 1999	181,774	7,418	216	-	- (41)	100	KT013210
Serro2	Serro (MG), 2005	184,572	6,794	209	-	41 (-)	97.14	KF179385

*a*: genome coverage was calculated using Tablet 1.17.08.17. Total number of reads was 33,710,700 (MI233), 35,756,458 (VI04), 34,215,020 (CG04), and 30,971,724 (ALEH2), with an average percentage of mapping > 90%; *b*: the number of single-nucleotide polymorphisms (SNPs) was determined using Base-by-base and is expressed in relation to CTGV-CM01 or Serro2 virus (in parentheses); *c*: identity matrix was calculated using CLC version 7.9.1 and is expressed in relation to CTGV-CM01 or Serro2 virus (in parentheses). ITR: inverted terminal repeat; ORFs: open reading frames.

**Fig. 2:**
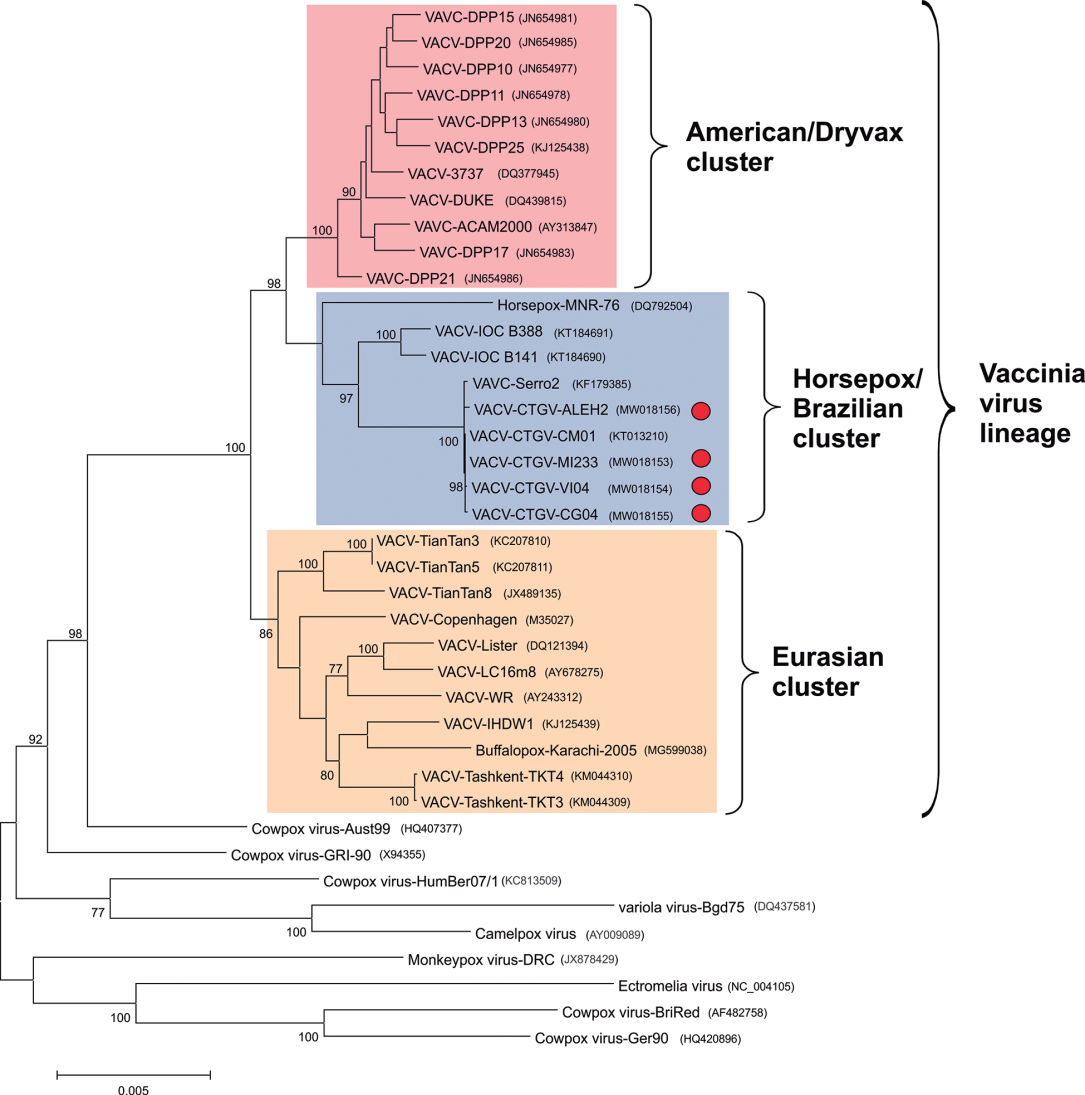
phylogenetic inference of Cantagalo virus (CTGV) isolates. A multialignment of the conserved region of 40 orthopoxvirus genomes was generated by Mafft vs 7.0 and used to construct a maximum likelihood tree using MEGA 6, opting for the kimura-2p model of substitution and a uniform rate. Numbers indicate the percentage of 1,000 replicates of bootstrap support. The scale bar indicates the number of substitutions per site. Coloured boxes indicate the three main clusters in the vaccinia lineage. The isolates sequenced in this work are indicated with red circles. Genbank accession numbers are indicated following the virus name.

**Fig. 3:**
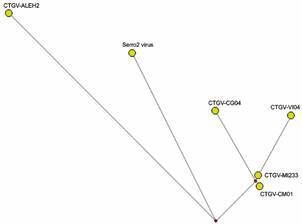
median-joining network of the sequences of Cantagalo virus (CTGV) isolates. The whole genomes of CTGV isolates CM01, MI233, VI04, CG04, ALEH2, and Serro2 virus were aligned using Mafft vs. 7.0, and the alignment was edited using Jalview version 2.11.1.1 to select sites with sequence variations. The resulting alignment was used as the input for Network version 5.0.1.1 software (https://www.fluxus-engineering.com/) to generate a median-joining network using default parameters. Branch length is proportional to the number of accumulated single-nucleotide polymorphisms (SNPs). The small red nodes (median vectors) indicate unsampled or extinct common ancestors.

**Fig. 4:**
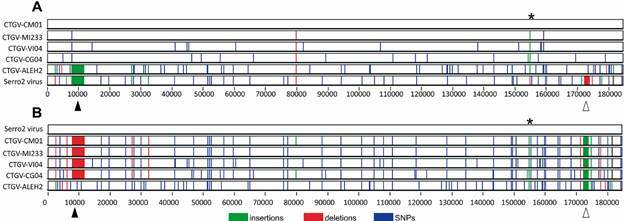
visualisation of the nucleotide substitutions, deletions, and insertions distributed along the genomes of Cantagalo virus (CTGV) isolates. The whole genomes of CTGV isolates CM01, MI233, VI04, CG04, ALEH2, and Serro2 virus were aligned using Mafft vs. 7.0, and the alignment was visualised using Base-by-base software (https://4virology.net/). Sequence variations per site single-nucleotide polymorphisms (SNPs) and insertions and deletions (INDELs) were highlighted in reference to the genome of CTGV-CM01 (A) or Serro2 virus (B). Asterisks indicate insertions in the A51R gene detected in some genomes. Black arrowheads indicate a 3.7 kb insertion shared by the genomes of CTGV-ALEH2 and Serro2 virus (A) or deletions shared by the genomes of CTGV-CM01, CTGV-MI233, CTGV-VI04, and CTGV-CG04 (B). White arrowheads indicate the 1.5 kb deletion unique to Serro2 genome (A), which is reflected as insertions in the other genomes when Serro2 virus is used as the reference (B).

The median-joining network explores the relatedness of the field isolates of VACV in greater detail and zooms in on the SNP-based distances among them (Fig. 3). We can observe that CTGV-ALEH2 is the most distant isolate, which is supported by the sequence identity scores (Table). The genome-wide screening for variant sites shown in Fig. 4 expands this analysis and shows the distribution of SNPs and INDELs along the whole genomes. Even for the three closest isolates (MI233, VI04, and CG04), the pattern of SNPs differs from each other. However, CTGV-VI04 has the same pattern of CTGV-MI233 with an additional 11 substitutions (Fig. 4A). When the genomes of all five CTGV isolates were analysed against the genome of Serro2 virus, we observed a pattern of SNPs and INDELs, totaling 31 alteration groups, that are shared by all CTGV isolates (Fig. 4B). Interestingly, even six years apart from CTGV-CM01, CTGV-ALEH2 accumulated only 62 SNPs in its genome (Fig. 4A, Table). However, 81 SNPs were detected when isolate CTGV-ALEH2 was compared with Serro2 virus (Fig. 4B, Table). A descriptive analysis of all sites with sequence alterations in relation to the CTGV-CM01 genome, indicating nucleotide position and targeted ORF, is provided in the Supplementary data (Table).

The most striking difference observed in the base-by-base profile is an insertion of 3,753 bp in the left end of the genomes of CTGV-ALEH2 and Serro2 viruses (Fig. 4, black arrowhead). The insertion corresponds to the full-length C9L gene and fragments of the ankyrin-repeat CPXV-77kDa gene that are missing in the genomes of CTGV isolates CM01, MI233, VI04, and CG04. In the case of C9L gene, the genomes of these four CTGV isolates preserve a fragment corresponding to the first 579 bp of the 1,905-bp intact gene. A second deletion of 1,458 bp was detected only in the genome of Serro2 virus, and corresponds to part of the genes B16R and B17L, which are truncated in Serro2 virus only (Fig. 4, white arrowhead).

The impact of such SNPs and INDELs in the dissemination and virulence of the CTGV isolates is unknown. The CPXV 77 kDa gene, despite being present in the genomes of Serro2 virus and CTGV-ALEH2, it is not as a full gene, but fragmented, similar to all other VACV genomes, in which this gene is either fragmented or absent.^26^ On the other hand, C9L is intact in several VACV genomes and has been recently shown to have a role in the evasion of the type-I interferon response.^27^ Therefore, it will be important to determine the genetic diversity of other VACV isolates associated with outbreaks in other regions of Brazil and investigate the prevalence of both genome patterns (with and without the C9L deletion) in this country.

The clustering of the 3 RJ isolates and VI04 suggests interrelated outbreaks, which is supported by the proximity of the events and of the localities. Interestingly, the CG04 isolate is in an intermediate position regarding year of the outbreak and distance (approximately 150 km) from MI233 (RJ) and ALEH2 (ES). However, ALEH2 is more distant genetically from CG04 than MI233 is. Additionally, the deletion in the region of the CPXV 77 kDa and C9L genes present in the ALEH2 genome is not shared with the 3 RJ and VI04 isolates, but is shared with Serro2 virus, which was collected in the previous year but approximately 500 km apart. However, although sharing this structural feature, ALEH2 and Serro2 genomes have accumulated 81 SNPs. Taken together, our data suggest a different event of virus spread into ES, not originated from the Northwestern region of RJ and not directly originated from Serro2, reflecting the complex genomic diversity of CTGV isolates related to the first years of the outbreaks that emerged in the Southeastern region of Brazil in 1999. These studies will lead to further investigation of the focal point of VACV dissemination in Brazil, and the biological and virulence diversity of the isolates.
