# Supplementary material for: Genomic diversity of vaccinia virus strain Cantagalo isolated in southeastern Brazil during the early years of the outbreak, 1999-2006
Source: Mem Inst Oswaldo Cruz. 2021 Feb 3;115:e200521. doi: 10.1590/0074-02760200521 (PMC7849178; doi:10.1590/0074-02760200521)
Supplement: Supplementary file 1 [file 1678-8060-mioc-115-e200521-s.pdf]

TABLE

Single-nucleotide polymorphism (SNP) analysis for CTGV-MI233, CTGV-VI04, CTGV-CG04, CTGV-ALEH2, and Serro2 virus genomes in relation to the genome of CTGV-CM01

| Nucleotide position in CTGV-CM01 genome | SNP | CTGV-MI233 | CTGV-VI04 | CTGV-CG04 | CTGV-ALEH2 | Serro2 virus | VACV-Cop ortholog | Product |
|---|---|---|---|---|---|---|---|---|
| 690 | G/A | | | | | X | Intergenic region | - |
| 4027 | C/T | | | | X | | C12L | serine protease inhibitor 1 |
| 4717 | A/T | | | | X | X | C12L | serine protease inhibitor 1 |
| 4756 | C/T | | | | X | | C12L | serine protease inhibitor 1 |
| 5917 | G/A | | | X | | | C10L | truncated IL-1 receptor antagonist |
| 8067 | A/T | | | | X | | Intergenic region | - |
| 8686 | T/C | X | X | X | X | X | C9L | ankyrin like protein |
| 11467 | G/A | | X | | | | C5L | hypothetical protein |
| 12870 | C/T | | | | X | | C3L | secreted complement C3b/C4b-binding protein |
| 14237 | G/A | | | | | X | C2L | POZ/BTB kelch domain protein |
| 16681 | C/T | | | | | X | N2L | putative Bcl-2 protein |
| 22016 | G/A | | | | | X | K4L | nicking-joining enzyme |
| 24144 | G/A | | | | X | | K7R | putative Bcl-2 protein |
| 24620 | T/C | | | | X | X | F1L | putative Bcl-2 protein |
| 24988 | C/T | | | | X | | F2L | deoxyuridine 5'-triphosphate nucleotidohydrolase |
| 26914 | C/T | | | | | X | F4L | ribonucleotide reductase small subunit |
| 28025 | C/T | | | | X | | F5L | 36 kDa major membrane protein |
| 28488 | C/T | | | | X | | F5L | 36 kDa major membrane protein |
| 33366 | G/A | | | | X | | F12L | KLC-like protein |
| 34631 | C/T | | | | X | | F12L | KLC-like protein |
| 37819 | C/T | | | | | X | F17R | lateral body phosphoprotein VP11 |
| 37911 | C/T | | X | | | | F17R | lateral body phosphoprotein VP11 |
| 38942 | G/A | | | | X | | E1L | poly(A) polymerase large subunit |
| 40627 | G/A | | | | X | | E2L | WV assembly/interaction with F12 |
| 41556 | G/A | | | | X | | E2L | WV assembly/interaction with F12 |
| 41893 | A/C | | X | | | | E3L | PKR inhibitor |
| 43254 | G/A | | | X | | | E5R | virosome component protein |
| 43979 | G/A | | | | | X | E5R | virosome component protein |
| 46281 | G/A | | | X | | | E7R | EV myristylated soluble protein |
| 48241 | C/T | | | | X | | E9L | DNA polymerase |
| 48343 | C/T | | | | X | | E9L | DNA polymerase |
| 48369 | C/T | | | | | X | E9L | DNA polymerase |
| 48468 | C/T | | | | X | | E9L | DNA polymerase |
| 49117 | G/A | | | | | X | E9L | DNA polymerase |
| 49530 | C/T | | | | | X | E9L | DNA polymerase |
| 52481 | C/T | | | X | | | O1L | hypothetical protein |
| 52904 | C/T | | | | X | | O1L | hypothetical protein |
| 53542 | T/C | | | | X | X | O2L | non-essential glutaredoxin 1 |
| 56681 | T/C | | | | X | X | I4L | ribonucleotide reductase large subunit |

CC BY

| Nucleotide position in CTGV-CM01 genome | SNP | CTGV-MI233 | CTGV-VI04 | CTGV-CG04 | CTGV-ALEH2 | Serro2 virus | VACV-Cop ortholog | Product |
|---|---|---|---|---|---|---|---|---|
| 57427 | G/A | | X | | | | I4L | ribonucleotide reductase large subunit |
| 61359 | G/A | | | | X | | I8R | DExH-nucleoside triphosphate phosphohydrolase II |
| 61925 | G/A | | | | | X | I8R | DExH-nucleoside triphosphate phosphohydrolase II |
| 62998 | C/T | | | X | | | G1L | insulin metalloproteinase-like protein |
| 72780 | G/A | | | | | X | L3L | internal virion protein |
| 74059 | G/A | | | | | X | L5R | integral component of virus entry/fusion complex |
| 79086 | C/T | | X | | | | J6R | DNA-dependent RNA polymerase subunit RPO147 |
| 81100 | C/T | | | | X | | J6R | DNA-dependent RNA polymerase subunit RPO147 |
| 85045 | G/A | | | | | X | H4L | RNA polymerase associated protein RAP94 |
| 86224 | G/A | | | X | | | H5R | viral late transcription factor VLTF-4 |
| 91241 | G/A | | | | X | | D3R | virion core protein |
| 91244 | G/A | | | | | X | D3R | virion core protein |
| 92377 | G/A | | | | X | | D5R | NTPase |
| 97836 | G/A | | | | | X | D8L | GAG-binding MV membrane protein |
| 100240 | C/T | | | | X | | D11L | ATPase |
| 100523 | G/A | | | | X | | D11L | ATPase |
| 101753 | G/A | | | | | X | D12L | mRNA capping enzyme small subunit |
| 103835 | G/A | | X | | | | D13L | crescent/IV scaffold protein |
| 104916 | C/T | | | | | X | A2L | late gene transcription factor VLTF-3 |
| 107639 | C/T | | | X | | | A4L | 39 kDa core protein |
| 107802 | G/A | | | | | X | A4L | 39 kDa core protein |
| 114940 | G/A | | | X | | | A10L | major core protein 4a precursor |
| 115387 | T/C | | | | X | X | A10L | major core protein 4a precursor |
| 116371 | C/T | | | | X | | A11R | viral membrane biogenesis protein |
| 118715 | C/T | | | X | | | A16L | integral component of the virus entry/fusion complex |
| 123465 | G/A | | | | X | | A20R | DNA polymerase processivity factor |
| 125100 | G/T | | | | X | | A23R | intermediate gene transcription factor VITF-3 45kDa large subunit |
| 125288 | T/C | | | | X | X | A24R | DNA-dependent RNA polymerase subunit RPO132 |
| 125908 | C/T | | | | X | | A24R | DNA-dependent RNA polymerase subunit RPO132 |
| 126793 | G/A | | | | X | | A24R | DNA-dependent RNA polymerase subunit RPO132 |
| 128635 | C/T | | | | X | | A25L | cowpox A-type inclusion protein |
| 131347 | G/A | | | | | X | A25L | cowpox A-type inclusion protein |
| 134634 | C/T | | | | X | | A28L | integral component of the virus entry-fusion complex |
| 134986 | G/A | | X | | | | A29L | DNA-dependent RNA polymerase subunit RPO35 |
| 136742 | G/A | | | | X | | A32L | DNA packaging protein |
| 140171 | G/A | | | | X | | A37R | hypothetical protein |
| 140247 | C/T | | | X | | | A37R | hypothetical protein |
| 140474 | G/A | | | | | X | Intergenic region | - |
| 144589 | C/T | | | | X | | A42R | profilin-like protein |
| 146124 | C/T | | | | X | | A44L | 3-beta-hydroxysteroid dehydrogenase |
| 146154 | G/C | | | | X | X | A44L | 3-beta-hydroxysteroid dehydrogenase |
| 146533 | C/T | | | | | X | A44L | 3-beta-hydroxysteroid dehydrogenase |

| Nucleotide position in CTGV-CM01 genome | SNP | CTGV-MI233 | CTGV-VI04 | CTGV-CG04 | CTGV-ALEH2 | Serro2 virus | VACV-Cop ortholog | Product |
|---|---|---|---|---|---|---|---|---|
| 147559 | G/A | | | | | X | A46R | putative Bcl-2 protein |
| 148260 | C/T | | X | | | | A47L | hypothetical protein |
| 150361 | G/A | | | | X | | A50R | DNA ligase |
| 151768 | G/A | | | | X | | A51R | hypothetical protein |
| 152173 | G/A | | | | | X | A51R | hypothetical protein |
| 153550 | G/A | | | | X | | A54L | hypothetical protein |
| 153567 | C/T | | | | X | | A54L | hypothetical protein |
| 154383 | C/T | | | | | X | A55R | kelch-like protein |
| 154438 | G/A | | | X | | | A55R | kelch-like protein |
| 155144 | C/T | | X | | | | A55R | kelch-like protein |
| 155246 | G/A | | X | | | | A55R | kelch-like protein |
| 155842 | G/A | | | | X | | A55R | kelch-like protein |
| 156098 | G/A | | | | X | | A56R | hemagglutinin |
| 156123 | G/A | X | X | | X | | A56R | hemagglutinin |
| 156632 | A/G | | | | X | X | A56R | hemagglutinin |
| 156713 | G/A | | | | X | | A56R | hemagglutinin |
| 156950 | G/A | | | | | X | Intergenic region | - |
| 157064 | C/T | | | | X | | A57R | guanylate kinase |
| 161072 | G/A | | | | | X | B4R | ankyrin-like protein |
| 161503 | C/T | | | | | X | B4R | ankyrin-like protein |
| 163430 | G/A | | | | X | | B6R | ankyrin-like protein |
| 165238 | G/A | | | | | X | Intergenic region | - |
| 170243 | G/T | | | | X | | B17L | hypothetical protein |
| 171938 | G/A | | | | X | | B18R | ankyrin-like protein |
| 174151 | T/C | | | | X | X | Intergenic region | - |
| 175858 | C/T | | | X | | | C10L | truncated IL-1 receptor antagonist |
| 177019 | G/A | | | | X | | C12L | serine protease inhibitor 1 |
| 177058 | T/A | | | | X | X | C12L | serine protease inhibitor 1 |
| 177122 | G/A | | | | X | | C12L | serine protease inhibitor 1 |
| 177748 | G/A | | | | X | | C12L | serine protease inhibitor 1 |
| 181085 | C/T | | | | | X | Intergenic region | - |
| 181148 | C/T | | | | X | | Intergenic region | - |

Twenty-one genes, marked in red, have SNPs in the genome of more than one clinical isolate when compared with CTGV-CM01. All clinical isolates share a T/C change in the C9L gene when compared with CTGV-CM01 (position 8686 in the CTGV-CM01 genome). SNPs in C10L, E7R, G1L, H5R, A10L, and A16L are unique to the CTGV-CG04 genome. SNPs in C5L, E3L, D13L, A29L, and A47L are unique to the CTGV-VI04 genome. CTGV: Cantagalo virus; VACV: vaccinia virus.
